# Stress Varies Along the Social Density Continuum

**DOI:** 10.3389/fnsys.2020.582985

**Published:** 2020-10-20

**Authors:** Jay Love, Moriel Zelikowsky

**Affiliations:** Department of Neurobiology and Anatomy, School of Medicine, University of Utah, Salt Lake City, UT, United States

**Keywords:** social isolation, stress, social density, crowding, brain modifications

## Abstract

Social stress is ubiquitous in the lives of social animals. While significant research has aimed to understand the specific forms of stress imparted by particular social interactions, less attention has been paid to understanding the behavioral effects and neural underpinnings of stress produced by the presence and magnitude of social interactions. However, in humans and rodents alike, chronically low and chronically high rates of social interaction are associated with a suite of mental health issues, suggesting the need for further research. Here, we review literature examining the behavioral and neurobiological findings associated with changing social density, focusing on research on chronic social isolation and chronic social crowding in rodent models, and synthesize findings in the context of the continuum of social density that can be experienced by social animals. Through this synthesis, we aim to both summarize the state of the field and describe promising avenues for future research that would more clearly define the broad effects of social interaction on the brain and behavior in mammals.

## Introduction

**Figure 1:**
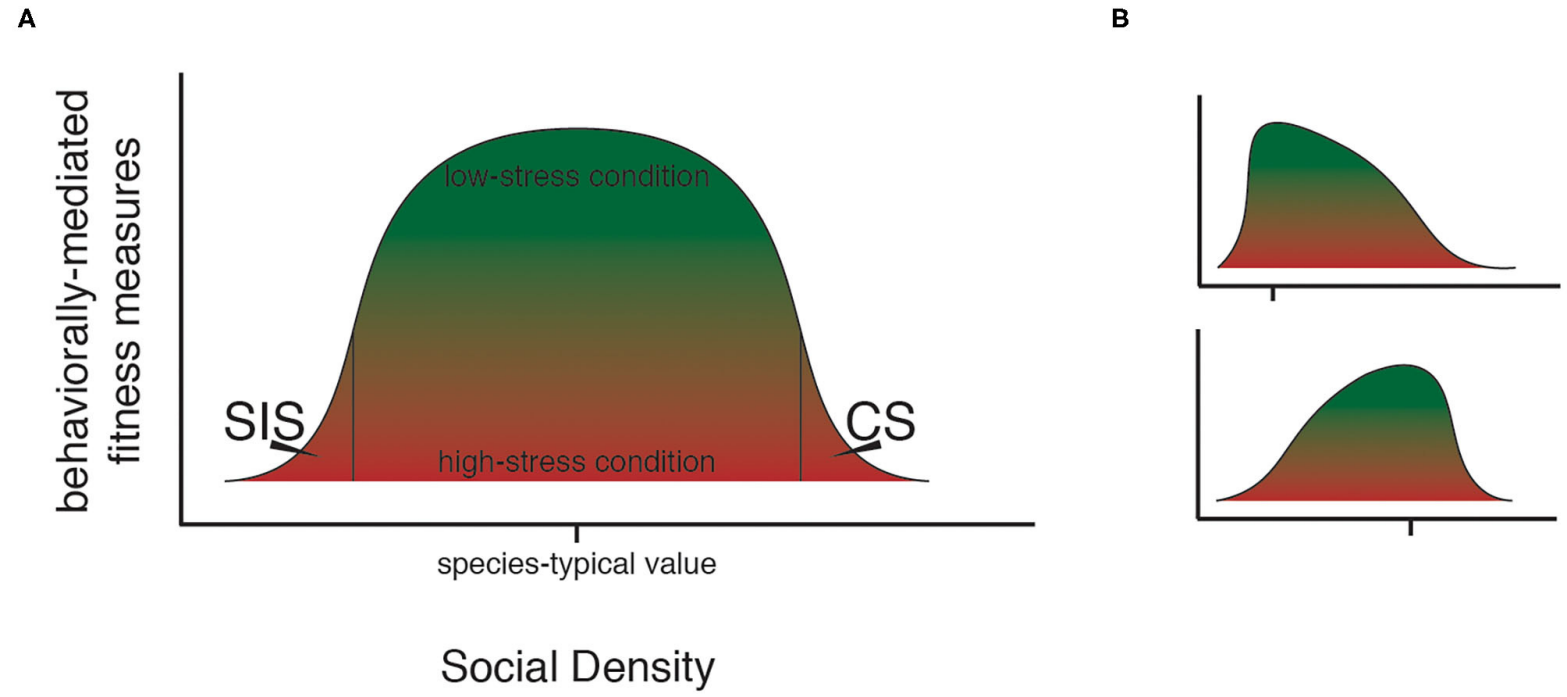
The social density continuum. As the magnitude of social density rises, different forms of stress occur in a population. **(A)** Through the action of social density-related stress, a curve-shaped upper bound for possible fitness states exists within a typical population. At species-typical levels of social density, individuals in a population may experience fitness states ranging from low (red) to high (green), while at extreme levels of social density, only low fitness is possible. **(B)** The shape of the bounding curve for possible states in social density-fitness space can be expected to vary based on taxon-specific features of the social system such as degree of social cohesion and mating system, as in these hypothetical examples. Hash marks show species-typical social density levels. Top panel shows an example for a species that achieves maximum fitness at comparatively low social density (i.e., social organization tends toward small social groups); bottom panel shows an example for a species that achieves maximum fitness at comparatively high social density (e.g., taxa that benefit from large social groups). Species, including model taxa, which differ in social organization can be expected to be comparatively more- or less- tolerant of extremes in social density.

**Figure 2:**
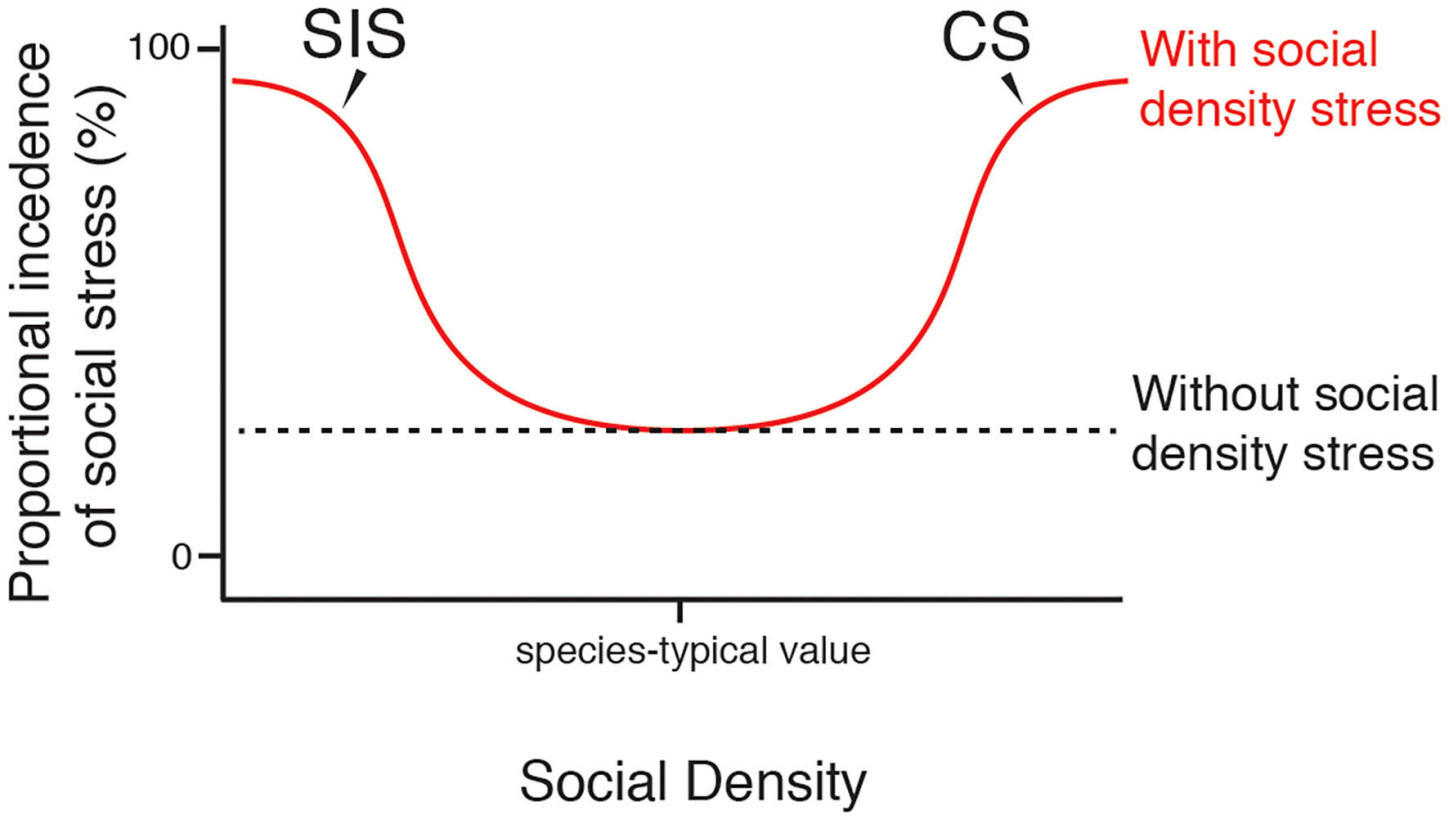
Proportional prevalence of social stress varies with social density. Curves show the proposed general effect of SIS and CS on the proportional prevalence of stress that is induced by the social environment and social activities in a population. At species-typical levels of social density, SIS and CS occur infrequently. At levels of social density far from species-typical values, greater proportions of the population will experience social stress via the action of SIS and CS. Individuals within a population will vary in their resilience to extreme levels of social density, thus social stress may not reach complete saturation.

Stress is a fascinating topic of research, largely due to the variable and enigmatic manner in which stress manifests itself across species and between individual animals. Stress can have context- and experience-dependent effects, and it is both essential and detrimental. Drawing from a rich history of stress research, there has been a theoretical formulation of stress as existing on a continuum, with an optimum level of stress positioned between under- and overstimulation (Selye, 1976; Sapolsky, 2015). In line with this, it can be considered that a single stress-inducing stimulus may exist on a continuum, producing a roughly bell-shaped curve, wherein an optimum magnitude of the stimulus could provide a benefit to the individual while either extremely low or high levels impart costs. Here, we apply this theoretical model to the understanding of psychogenic stress in response to social density (Figures 1, 2).

**Figure 3:**
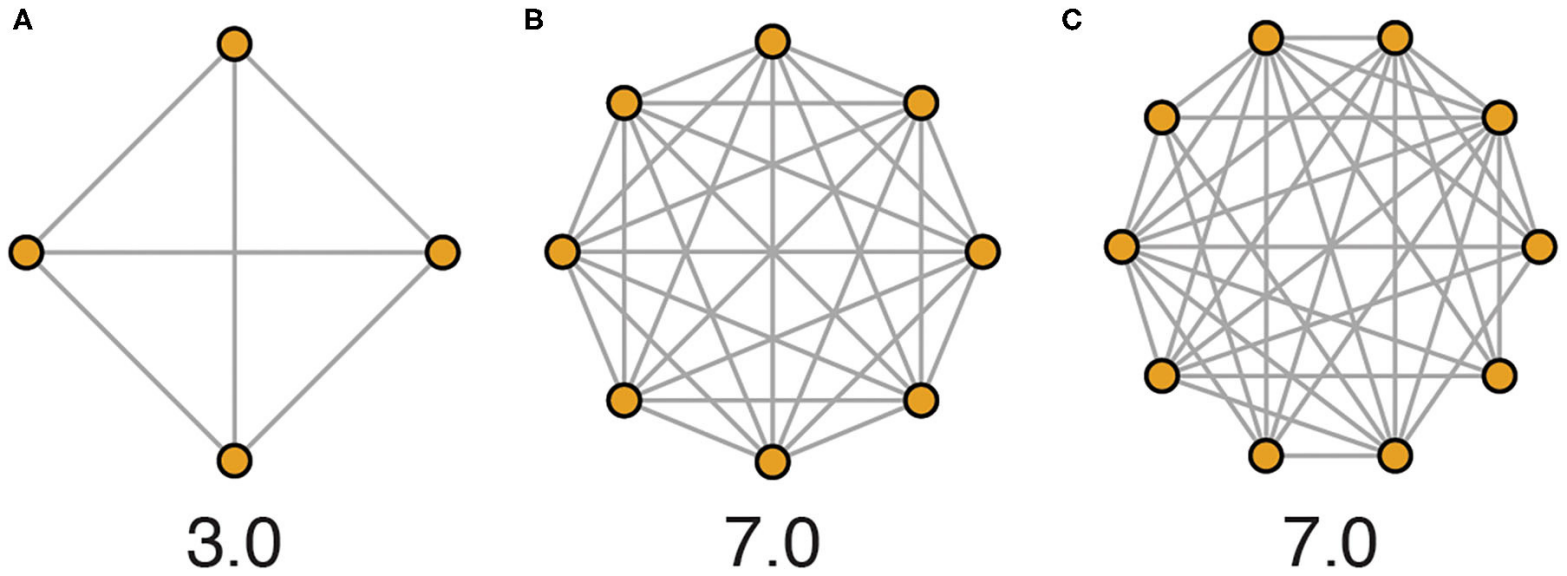
Network illustrations depicting hypothetical social density across housing schemes evaluated over 1 day. **(A)** In a typical laboratory scheme, mice are housed 4 per cage, and each mouse encounters every other mouse every day. Here, 12 unique social encounters occur per day among 4 animals, equating to a social density of 3.0 encounters per individual per day. **(B)** In a more densely populated housing scheme of 8 mice per cage, social density would be calculated as 56 encounters per day among 8 animals, equating to a social density of 7.0 encounters per individual per day. **(C)** In more complex environments or with higher population numbers, all members of a population are less likely to come into social contact with all other members every day, and so accurately calculating actual average social density requires more detailed data (e.g., 70 unique encounters among 10 individuals, social density again equals 7.0, as shown).

“Social density” as we use it here refers to the average conspecific encounter rate in a population of animals. While social density could be defined through numerous approaches that variably include measures of physical space, we advocate for a framework that emphasizes the social environment by quantifying the average number of unique social encounters in a given population that occur per unit of time, a concept equivalent to the average degree of a network (Figure 3). In this way, physical area and resource availability of the habitat are indirectly related to social density.

Under species-typical levels of social density, social stress occurs. However, this stress is likely to be well-tolerated, perhaps even an integral part of the social system which has evolved alongside the taxon in question. In such situations, neural regulation of the stress response typically operates under conditions to which it is well-equipped to handle. In humans, social stress can be triggered by interpersonal encounters, argument, and fighting. Similar patterns exist in rodent models, where social stress is often studied in the context of mating, aggression, and the establishment of social hierarchy. Each of these behaviors can be enhanced at the individual level by regulation of the HPA axis (Korte et al., 2005). Stressors in this category are often regular occurrences in the lives of animals, and they have the ability to adaptively guide behavioral development. However, when allostatic load, the strain on the body due to the physiological response to stress (McEwen and Stellar, 1993), is chronically high, such stressors can inflict severe and persistent abnormal behavioral syndromes (McEwen, 2000; McEwen and Wingfield, 2003; Tamashiro et al., 2005; Beery et al., 2020). Through allostasis, such social stressors together contribute to an overall increase in fitness, keeping an individual on track for success in its social environment (McEwen, 2000; Korte et al., 2005).

In contrast to moderate forms of social density and stress, mammals respond poorly to extremes in social abundance and density. In comparison to moderate levels, extremely low or high social density may constitute a situation for which the nervous system is not tuned to efficiently handle, especially when administered at chronic levels. Stress associated with such extremes, namely social isolation stress (SIS) and social crowding stress (CS), are well-known to induce chronic stress in animals (Gamallo et al., 1986; Hilakivi et al., 1989; Djordjević et al., 2003; Weiss et al., 2004; Lin et al., 2015). Indeed, these manipulations are some of the few that are truly chronic stressors, in that there is no reprieve from the stressor at any point (Zelikowsky et al., 2018a). In contrast, other models of chronic stress administer acute forms of stress repeatedly over time (e.g., social defeat; Berton et al., 2006; Keeney et al., 2006; Golden et al., 2011). Extended periods of continuous social isolation are known to induce an array of negative behaviors in humans and animal models, and the neural mechanisms that facilitate this change in behavior have recently been exciting topics of research. Chronic social crowding has been observed to induce other changes in behavior in several model systems, but less attention has been paid to understanding the neurobiological underpinnings of this form of stress. Importantly, both forms of extreme social density stress occur across the animal kingdom.

In society today, understanding the effects of variation in social density on the brain and behavior has become increasingly important. During the COVID-19 pandemic, many around the world are (at time of writing) confined to their own spaces for extended periods of time in order to reduce viral transmission (Anderson et al., 2020; Armitage and Nellums, 2020; He et al., 2020). In juxtaposition, major population centers have experienced historically high population growth rates that have led to dense living conditions for many others (Taylor, 2006; Buhaug and Urdal, 2013). To understand the way in which variation in the abundance and density of social interactions, irrespective of their specifics, directly influences the workings of the brain is an important goal for future research. Here, we review research on social stress, focusing on extremes in social abundance and density in rodent models.

An Allee effect is any mechanism that leads to a positive relationship between some individual fitness measure and the number or density of conspecifics in a population (Odum and Allee, 1954; Stephens and Sutherland, 1999; Stephens et al., 1999; Angulo et al., 2018). In this review, we describe how social-density-induced stress constitutes a mechanism through which individual fitness is contingent on population size and social density. Individual fitness is reduced by SIS and CS at extremely low and extremely high population densities relative to fitness at moderate population densities. Thus, this describes behaviorally-mediated positive- and negative-density dependence, including a de-facto Allee effect mechanism. With this acknowledgment, ecology may be able to inform future work in neurobiology and vice-versa.

## Low Density: Social Isolation Stress

Social species are defined by their conspecific interactions, which are often multidimensional, complex, and pervasive across lifetimes. Denied exposure to conspecific peers, social animals will behave abnormally. Research on social isolation can be divided into two broad categories based upon whether it focuses on the effect of isolation during development or during adulthood.

### Impact of Social Isolation During Development

Social isolation during early development has been shown to impart an anxiety-like phenotype and other behavioral changes in adult rodents (for review see Lapiz et al., 2003; Fone and Porkess, 2008; Mumtaz et al., 2018). Among the behaviors that are altered, many are social in nature. For example, one study found that developmentally-isolated male mice showed a decrease in adult social competence characterized by altered ultrasonic vocalizations (USVs) and increased male-male mounting behavior (Keesom et al., 2017). Another study found that developmentally isolated adult female mice take longer than socially-housed mice to learn an operant task, though after training they did not show reduced performance on a USV discrimination task (Screven and Dent, 2019). Notably, one study suggests that the effects of adolescent social isolation may be ameliorated by partial indirect social housing, holding promise for future study (Pais et al., 2019).

Focused investigations have been made to determine the neural mechanisms underlying these behavioral changes. In adolescent mice that have been socially isolated, differences arise in the network organization of the structural connectome relative to controls, such that measures of network structure such as modularity (the strength of the division of a network into modules) and small-worldness (the degree to which a network is organized into clusters) decrease, indicating greater homogeneity of connections (Liu et al., 2016). In particular, these alterations hinged on disruption of inter-hemispheric and inter-modular connections of the dorsolateral orbitofrontal cortex (Liu et al., 2016). In adulthood, studies have found developmental social isolation to be associated with reduced myelination, altered dendritic spine development, and reduced plasticity in the pre-frontal cortex [PFC; (Makinodan et al., 2012; Medendorp et al., 2018)], and changes in PFC connectivity (Hermes et al., 2011), especially regarding the circuit between the PFC and amygdala (Castillo-Gómez et al., 2017). Serotonergic fiber density in the inferior colliculus has also been shown to be reduced by developmental isolation (Keesom et al., 2018).

### Impact of Social Isolation in the Adult

Social isolation also has detrimental effects when experienced during adulthood. This is especially abundant in the elderly and disabled populations, but can occur at any phase of adulthood (Hawkley and Cacioppo, 2010). Adult chronic social isolation is associated with increased risk of death (House et al., 1988) and a suite of mental health issues in humans (Cacioppo et al., 2011). For these reasons, studies of chronic social isolation in model systems are apt to provide relevance for translation to humans.

Adult chronic SIS has been shown to induce a phenotype sharing characteristics with anxiety, depression, and social withdrawal in rats and mice (Scaccianoce et al., 2006; Liu et al., 2012; Ieraci et al., 2016), especially characterized by increased aggression (Allee, 1942; Valzelli, 1973). Given these findings, attempts have been made to dissect the neurobiological correlates of SIS-induced behavioral changes.

SIS is notable in that, despite strongly impacting behavior, it does not appear to increase plasma corticosterone (CORT) or brain monamines such as noradrenaline and dopamine, which are induced by other stressors (Hilakivi et al., 1989; Scaccianoce et al., 2006). For example, one study found reductions in allopregnanolone and 5a-dihydroprogesterne in SIS mice (Dong et al., 2001), and another found significant reductions in plasma corticosterone (Ieraci et al., 2016). Notably, this effect seems to differ across sexes: females exhibit higher CORT in isolation, while males undergoing the same conditions exhibit a mean reduction in plasma CORT (Brown and Grunberg, 1995).

**Figure 4:**
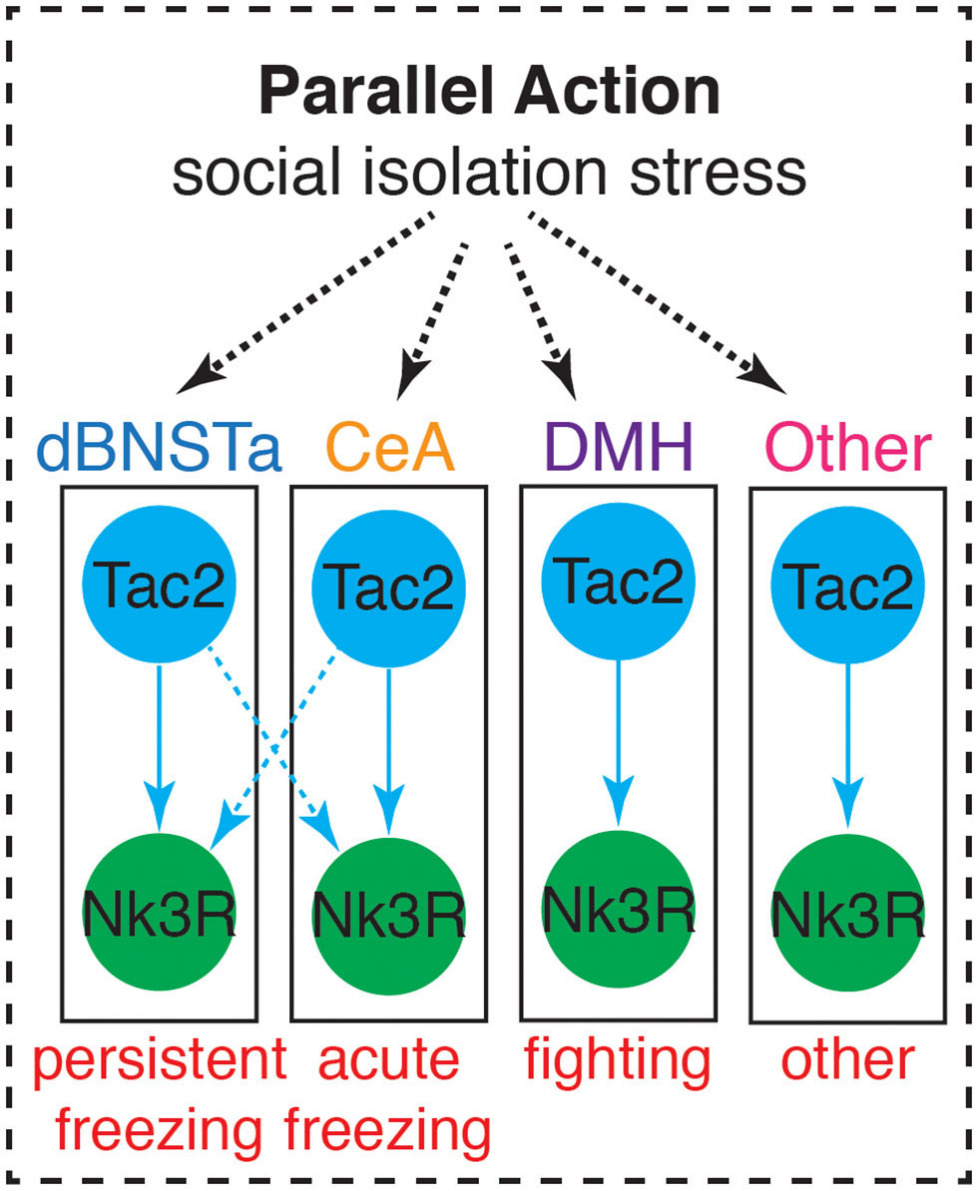
Tac2 exerts parallel action in multiple brain structures to influence behaviors associated with SIS. Adapted from Zelikowsky et al. (2018b).

Rather than showing more typical signs of acute stress, SIS has been associated with several neural changes. Included among these changes is decreased myelination in the PFC (Liu et al., 2012), a reduction of BDNF, mGluR1, and mGluR2 in the PFC, and lower immediate early gene expression in the PFC and hippocampus (Ieraci et al., 2016). Importantly, we recently discovered that SIS is accompanied by a brain-wide upregulation of the neuropeptide Tachykinin 2 (Tac2) (Zelikowsky et al., 2018b). Moreover, our data reveal dissociable roles for Tac2 signaling in the anterior dorsal bed nucleus of the stria terminalis (dBNSTa), dorsomedial hypothalamus (DMH), and the central amygdala (CeA) in the control of isolation-induced persistent fear, enhanced aggression, and acute fear, respectively. These data suggest that Tac2 exerts parallel action in multiple brain structures to control SIS and identify a key molecule in the control of social isolation (Figure 4).

### Factors Controlling the Effects of Social Isolation

Another key factor determining the extent to which social isolation is detrimental is the degree and duration in which an animal is isolated. For example, one study found that isolation for 2–5 days was associated with a performance increase in the forced swim test characterized by reduced immobility time, but isolation for 10–20 days was not (Hilakivi et al., 1989). In the same vein, a study of the effects of 24 h social isolation found that mice prefer to interact with a novel mouse after this short period of isolation (Matthews et al., 2016), which contrasts with the reduction in social interaction seen following longer, chronic forms of social isolation (Zelikowsky et al., 2018b).

The relationship between chronicity of social isolation and negative effects on the brain and behavior may also be evident in humans. While the negative feelings associated with chronic loneliness are well-studied (Hawkley and Cacioppo, 2010), research that investigates potential benefits of time spent alone is less common. Still, reports suggest that time spent alone can be emotionally beneficial, bringing feelings of freedom, creativity, and intimacy (for review see Long and Averill, 2003). One study found that humans experiencing brief social isolation felt increased high arousal negative affect and heightened salivary cortisol, as expected if isolation is detrimental, but also that these changes in high arousal affect were associated with a simultaneous increase in low arousal positive affect (Pauly et al., 2017). Importantly, this relationship differed with age: adults over 68 years old did not show increased cortisol with brief social isolation while younger groups did (Pauly et al., 2017). Similarly, another study found social isolation to have a detrimental effect on high-arousal affects but a restorative influence on low-arousal positive affect (Nguyen et al., 2018). That same study found that choosing to be alone in an unrestricted environment dramatically decreased measures of experienced stress, increased feelings of relaxation, and heightened high-arousal positive affect (Nguyen et al., 2018). With these findings in mind, defining the context- and chronicity- dependent role of social isolation on human health can be expected to be an important line of future research.

## High Density: Social Crowding Stress

When a population's size increases, changes in individuals' behaviors will arise as a result. For example, crowding produces a stressful increase in competition for limited resources, and increased aggression and territoriality are maintained in the offspring of crowded populations (Agrell et al., 1995). Early research has focused on determining the existence of individual-level population control mechanisms which might be manifested in behavioral change. Such mechanisms were hypothesized to be resource-independent and triggered by high social density. One line of study found that male mouse adrenal gland weight increased with increasing population density, presumably indicating an increase in adrenal function, and that the weight of the pituitary gland, seminal vesicles, and testes declines with increasing social density, suggesting decreased secretion of testicular androgens (Christian, 1961). Importantly, these findings also suggest that a greater absolute number of individuals in a population will trigger the observed changes regardless of the amount of space that population occupies (Christian, 1961).

Another line of early research used mice in semi-natural environments to evaluate the effects of density on behavior (Marsden, 1972). This research showed that a number of abnormal behavioral phenotypes emerged with increased population density among both males and females and that the emergence of these phenotypes was associated with a decline in typical behaviors found under less crowded conditions. At the highest population densities, phenotypes were characterized by extreme social withdrawal. This phenotype continued even after reintroduction of such individuals to non-crowded populations of mice raised under typical densities (Marsden, 1972).

Whether or not an adaptive explanation exists for the mechanisms at work, there appear to be significant behavioral and endocrine effects of social crowding occurring both during development and in adulthood. One study found that crowding during early development does not immediately increase CORT, but that corticoadrenal responses to acute stress (startle and forced swim) were increased compared to non-crowded mice (Ortiz et al., 1985). Another study found effects of crowding on nociception regulation in developmentally-crowded mice, but their control condition was social isolation, rendering interpretation of their results difficult in the current context (Reiss et al., 2007). Interestingly, it appears that limited developmental crowding can be beneficial—not harmful—to mice, since one study found that nighttime crowding of adolescent mice reduces anxiety-associated behaviors (Ago et al., 2014). This finding that a degree of crowding is net-positive for the individual again bolsters the conceptualization of social density stress as a continuum and highlights the importance of chronicity in mediating the negative effects of crowding or isolation.

In adulthood, the results of studies of social crowding stress sometimes conflict. In rats, crowding has been shown to induce increased CORT (Gamallo et al., 1986; Djordjević et al., 2003), but another study found that effects of crowding are sex-specific: females show no change in CORT between group-housed and crowded conditions, while males show a marked increase in CORT with crowding (Brown and Grunberg, 1995). In contrast to the above studies that find increased response to acute stress by developmentally-crowded mice, a series of studies found that crowding in adult rats significantly reduces HPA responsiveness to noradrenaline (Bugajski et al., 1994). Much less work on crowding has been done in mice than in rats, but one study found that crowding induced increased adiposity (but not weight gain) and mild anxiety-like behavior (but no increase in depression-like tendencies). Importantly, this study also found gene expression changes in the hypothalamus (Lin et al., 2015).

Fear less work (or research) has been conducted on the neural effects of CS than on other forms of social stress, with past research being primarily focused on behavior and the neuroendocrine response. Future research in this arena is encouraged. Such studies would help refine our understanding of the behavioral and neural impacts of social crowding stress and seek unifying explanations for these findings.

## Typical Density: Acute, Fleeting, Heterogenous, Stress

In comparison to extremes in social density, species-typical social densities facilitate an increase in fitness (Lacey and Sherman, 2007; Silk, 2007). Indeed, empirical and theoretical work portrays social stressors at typical social density as being net-positive for the individual when they are administered acutely, contributing positively to adaptive social dynamics (McEwen, 2000; McEwen and Wingfield, 2003; Korte et al., 2005). Social stress at typical densities is a well-studied subject that has been reviewed thoroughly and recently (Tamashiro et al., 2005; Beery et al., 2020). Briefly, animals in conspecific groups experience social stress in the context of interactions such as fighting, courtship, and territory defense. These stressors are often fleeting or avoidable and vary in their abundance and magnitude across time. When administered chronically, these forms of stress can induce behavioral alterations [see literature on chronic social defeat (Berton et al., 2006; Keeney et al., 2006; Golden et al., 2011) and allostatic overload (McEwen and Wingfield, 2003)], but chronic loads are infrequent in average natural populations. Rather, a suite of theoretical and empirical studies have established that such social stressors facilitate the establishment of taxon-specific social structures and social dynamics that together exert a positive influence on individual fitness (for review see Korte et al., 2005).

Social taxa differ in their typical social density. *Mus musculus* and *Rattus norvegicus*, the two species from which laboratory strains of the common mouse and rat models are derived, have highly flexible social systems which allow living in up to 1,000 times higher densities when resource availability is high than when resources are sparsely distributed (Berdoy and Drickamer, 2007). In these taxa, the tendency to live in social groups varies along with population density (Berdoy and Drickamer, 2007), thus, realized individual-level social density of wild populations varies dramatically. Humans, on the other hand, have been described as having evolved to live in tightly-knit social and kin groups (Hawkes et al., 1998; Kaplan et al., 2009). Though a degree of variation occurs, deviation from the group-living habit amongst our own species is infrequent (Boyd et al., 2012). The social density thresholds and phenotypic effects associated with the onset of SIS and CS are likely to show interspecific variation that scales with both typical social density and the flexibility of the taxon-specific social organization (Figure 1B). Accounting for this variation in taxon-specific typical social density should be a priority for future research, as should identifying the possible existence of rules that govern the relationship between SIS and CS social density thresholds and species-typical social density. Indeed, the alignment between individual-level social density stress thresholds in humans and those in model taxa could hold relevance for the direction of research in the field.

Understanding social stress at typical densities may partially explain findings of SIS research. For example, individual differences in hierarchy position could introduce individual variation in the response to SIS. When group housed, both male and female mice form social hierarchies (Van Den Berg et al., 2015; Varholick et al., 2019; Williamson et al., 2019), and top-ranking positions are associated with reduced plasma CORT, decreased initiation of aggression, and increased reception of aggression (Louch and Higginbotham, 1967; Williamson et al., 2019; but see cases where CORT in top-ranking positions does not differ from that of lower ranks: Schoech et al., 1997; Pravosudov et al., 2003; Poisbleau et al., 2005). Hierarchy formation is typically associated with increased stress for all members, which can be reduced after a hierarchy becomes established (Beery et al., 2020). Results of studies that find changes in stress under social isolation may be clarified by accounting for social rank of individuals. In other words, short-term social isolation may be a relief for individuals that had a high-stress rank in group housing, while individuals from low-stress ranks may experience an increase in stress when moved from group to isolation housing.

In addition, the increased aggression widely observed after social reintroduction of SIS-treated mice (Valzelli, 1973) may be explained by the innate territoriality of male mice. Mice and rats have flexible social systems. In most wild populations, male mice hold territories and defend them from other males (Berdoy and Drickamer, 2007). This territorial aggression is reduced in forced group housing, where social hierarchies form, but subordinate males increase aggression when more space is made available in apparent attempts to establish territory (Beery et al., 2020). When a male is moved into isolation, it may be that that animal establishes itself as the dominant territory holder. When it is moved back into group housing, increased aggression may reflect the animal's newfound de-facto territorial dominance. In this way, a behavioral change widely reported as being atypical or maladaptive may be readily explained by the workings of the social system of mice. Understanding the effects of SIS on the brain and behavior in the context of the social system of the model organism should inform future research in the field.

While more work is necessary to form strong conclusions from the body of work on CS, the combined results may be explained by the possibility that crowding amplifies or exaggerates individual-level social stresses that occur under typical group-housed conditions. It has been suggested that disrupting social hierarchy can cause reduced neurogenesis in the hippocampus and altered social preferences via a preference for known over novel conspecifics (Opendak et al., 2016). Chronic social disruption has also been shown to induce anxiety and abnormal social behavior, which can be vertically inherited (Saavedra-Rodríguez and Feig, 2013). It is possible that social crowding could disrupt typical hierarchical organization by overloading the social mechanisms by which it forms, thereby inducing atypical social behavior and associated effects on the brain of group members. While this possibility deserves further testing, some support is garnered from past work. For example, one study which found that reducing group size increased overall hierarchy stability and decreased the time to hierarchy establishment in rats (Becker and Flaherty, 1968). Another found that angelfish hierarchies are less stable at higher group sizes (Ang and Manica, 2010). In addition, theoretical work has shown that increased group sizes decreases the likelihood of linear hierarchies (Mesterton-Gibbons and Dugatkin, 1995). It could also be argued that SIS constitutes de-facto hierarchy disruption, so some of the observations of socially isolated animals could be products of an active disruption, rather than of passive hierarchy absence.

Further behavioral examination of stress under typical social densities to explore these and other possibilities should prove very fruitful toward building a more complete understanding of the way that the brain and behavior respond to variation in social density. Additionally, focusing research efforts on common evolutionarily and ecologically important behaviors will likely be informative for stress research in model systems, as has been witnessed with the influential formulation and use of the predatory imminence scale in research on memory and fear (Fanselow and Lester, 1988; Perusini and Fanselow, 2015). For example, clarifying the behavioral algorithms (Hein et al., 2020) and neural mechanisms that guide such common behaviors as mating and foraging (Kolling et al., 2012) will be likely to contextualize findings of research on SIS and CS, stressful conditions under which these common important behaviors are disrupted.

## Discussion

In social animals, there is a relationship between social density and behavior such that moderate levels of density are beneficial and extreme levels of density are detrimental to an animal's behavior (Figure 1). While some individuals may experience heightened stress and reduced fitness under species-typical social density, the population average fitness will be higher under moderate than under extreme social density. In this framework, social density functions as a stressor, with extremely high and low levels of social density eliciting stress of different forms. Defining this curve for different social species will be an important goal of future research with interest in translation to human health—the shape of this curve for taxa with social systems most similar to our own may provide interesting application to our own lives and to the development of treatments for injuries inflicted by social density-associated stress.

As with any discussion of stress, it is important to recognize that “stress” has had many definitions over the years. Here, we have considered the potential effects of social density-related stress not only as any HPA-axis regulated neuroendocrine stress response and its dysregulation, but also as the behavioral and neural changes that could be either primary or secondary responses to psychogenic stress. Furthermore, we discuss SIS and CS as imparting limits on fitness in a population. To what degree these limits are the result of direct or indirect effects of stress is an important consideration, and disentangling cause from effect regarding the stress-phenotype components and population-level effects described in this review will be an interesting direction for future research.

While we use “social density” to refer to population-level average social encounter rate (Figure 3), social density also manifests at the individual level. In typical laboratory settings, social density is strictly controlled, and each individual is likely to experience the same realized social density. In natural populations, however, individuals have the potential to experience radically different social density, and this variation can be amplified by increased habitat complexity and the social system. Heterogeneity in the environment is likely to reduce the encounter rate among population members and increase individual variation in realized social density. Varying degrees of social group cohesion and other features of the social system can similarly contribute to increased variation in realized social density of individuals in a population. It follows that with this increased variation comes altered population-average social density and an increased likelihood for individuals to experience extremes in social density, along with SIS and CS.

We suggest a definition of social density that should provide utility to the field, but which relies on quantifying social “encounters,” the definition of which is also subject pitfalls. We propose considering an encounter to be any detection of a conspecific by an individual. While identification of encounters is made more difficult in taxa that interact via communicative modes that are relatively difficult to track (e.g., ultrasonic vocalizations, odors in rodents, etc.), opportunities abound for research into the neural and behavioral effects of social density experienced at different levels, in different contexts, and through different modes. Work that incorporates network science approaches to understand the effects of social density and social connectivity on the brain and behavior are likely to significantly advance the field (e.g., So et al., 2015; Williamson et al., 2016).

It should be recognized that effects of social isolation and social crowding have also been recorded in non-mammalian social species, including in insects (Wang et al., 2008; Lihoreau et al., 2009; Ueda and Wu, 2009; Stevenson and Rillich, 2013), fish (Halperin and Dunham, 1993; Brandão et al., 2015; Shams et al., 2015), and birds (Apfelbeck and Raess, 2008; Banerjee and Adkins-Regan, 2011). Notably, a recent study found immediate effects of social isolation in the expression of several immediate early genes in the forebrain of an avian species (George et al., 2019), a finding which has implications for the field of vocal learning, which historically relies on the isolation of animals (Love et al., 2019). It is difficult to determine at present how much of the human response to social density stressors is unique to humans; do we find similar responses among distantly-related taxa that show similarity in social systems, or does genetic relatedness play a more important role in determining how the CNS modulates behavior in response to social density? If the latter, then how evolutionarily conserved are the neural mechanisms that affect behavior in response to social density stress? Detailed investigations into these questions using powerful techniques under a behavior-first paradigm are likely to yield important insights into the specific neural mechanisms that are involved with extreme social density stress. Taking inspiration from the comparative study of the neural bases for other behaviors (Bullock, 1984; O'Connell and Hofmann, 2011a,b, 2012; Dulac et al., 2014; Barkan et al., 2017; Nieder, 2018; Paolino et al., 2020) and leveraging the abundant variation in social structure in diverse animal clades, future evolutionary comparative behavioral neuroscience research would likely be particularly fruitful to that end.

If SIS- and CS-induced behavioral changes are deeply evolutionarily conserved, then the abundant research on positive and negative density dependence could be informative for understanding these altered behavioral states and formulating hypotheses for future research. The combined behavioral changes related to SIS and CS are manifestations of the Allee effect and negative density dependence (Stephens and Sutherland, 1999; Stephens et al., 1999; Angulo et al., 2018). It is critical to point out that both SIS and CS differentiate themselves from many other forms of stress in that their effects on behavior are maintained for the remaining lifetime of the animal that experiences them and are thought to decrease fitness by altering courtship and mating behavior (Christian, 1961; Marsden, 1972). In this way, SIS and CS combine to create substantial density dependence, which would not exist if the effects of these stressors were more fleeting. While this recognition may appear to some as simple semantics, it is important to indicate the potential for neuroscience to investigate the neural substrates for evolutionarily and ecologically important behaviors (Krakauer et al., 2017). Likewise, research on the behavioral modification undergone during extremes in population density will be informative for building more accurate predictive models of population dynamics.

There is some evidence that short periods of social isolation may well be important for human mental health. Seeking solitude is a common experience for many (Long and Averill, 2003), and it has been proposed that a balance between aloneness and relatedness is pivotal to human health (Detrixhe et al., 2014). These observations may seem at odds with the often negative social connotations of being alone. However, such conflicting results regarding the emotional response to being alone may be explained by conditionality and context-dependency. Humans can be alone but not feel lonely, and they can feel lonely even though they are with others (Hawkley and Cacioppo, 2010). The difference between these mental states—uncomfortable loneliness vs. comforting solitude—is profound, given that they occur under the same social environment. The well-studied effects of social isolation on mental health in humans hinges on the prominence of chronic loneliness, not chronic solitude. In order to draw more accurate translation to humans, findings from model systems must be contextualized with these important results in mind. Indeed, we currently know very little about how periodic crowding and isolation may affect the brain and behavior of social animals. Future work on SIS that could characterize the emotional context- and dose-dependency of social isolation in terms of perceived solitude and perceived loneliness would constitute a meaningful addition to the field.

It may not be surprising that extremes of social density—those to which a given social species is not well-adapted—elicit abnormal behavior and neural activity respective to that typical of the species under normal social circumstances. Nonetheless, these models do allow for a simple manipulation to be used during powerful experimental investigation of the neural correlates of relevant behavior, and therefore may provide a fruitful system for understanding the organization of the brain. Furthermore, it seems remarkable that these forms of stress have the potential to induce lasting injury, their effects lingering long after an animal is returned to typical conditions. With an interest in human health, we must ask “why?” and “how?” these behavioral disorders arise. Further integrative study of social density stress resulting in a definition of the shape of the stress curve will allow for the identification of the levels at which these injuries become more likely to occur. With that information, we may be closer to being able to recognize the specific mechanisms by which these stress-induced disorders arise and, perhaps, prevent them.

## Author Contributions

All authors listed have made a substantial, direct and intellectual contribution to the work, and approved it for publication.

## Conflict of Interest

The authors declare that the research was conducted in the absence of any commercial or financial relationships that could be construed as a potential conflict of interest.
