# Erratum: Stress Varies Along the Social Density Continuum

**DOI:** 10.3389/fnsys.2021.782630

**Published:** 2021-10-05

**Authors:** Frontiers Production Office

**Affiliations:** Frontiers Media SA, Lausanne, Switzerland

**Keywords:** social isolation, stress, social density, crowding, brain modifications

Due to a production error, the Editor's note was included in the Editor's affiliation.

The publisher apologizes for this mistake.

The original article has been updated.

